# Supplementary material for: ER-mitochondria contacts promote mtDNA nucleoids active transportation via mitochondrial dynamic tubulation
Source: Nat Commun. 2020 Sep 8;11:4471. doi: 10.1038/s41467-020-18202-4 (PMC7478960; doi:10.1038/s41467-020-18202-4)
Supplement: Supplementary file 1 — Supplementary Information [file 41467_2020_18202_MOESM1_ESM.pdf]

Supplementary information

ER-mitochondria contacts promote mtDNA nucleoids active transportation via mitochondrial dynamic tubulation

Qin et al.

This file contains:

Supplementary Figures (1 to 10)

Supplementary Table 1

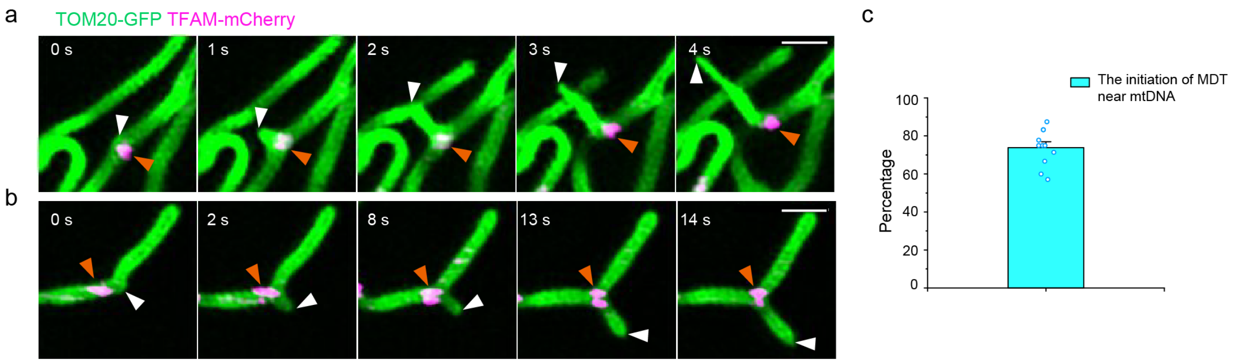

Supplementary Fig. 1 MDT are spatially linked to mitochondrial DNA nucleoids. **a** and **b)** Time-lapse images of Cos-7 cells expressing Tom20-GFP and TFAM-mCherry. White arrowheads mark the tips of tubules generated by the MDT processes. Orange arrowheads indicate the TFAM-mCherry–labeled nucleoids. Scale bar: 1 µm. **c)** Percentage of MDT in Cos-7 cells spatially linked to nucleoids (n=59 MDT events from 10 cells examined over three independent experiments). Data are presented as mean ± SEM. Source data are provided as a Source Data file.

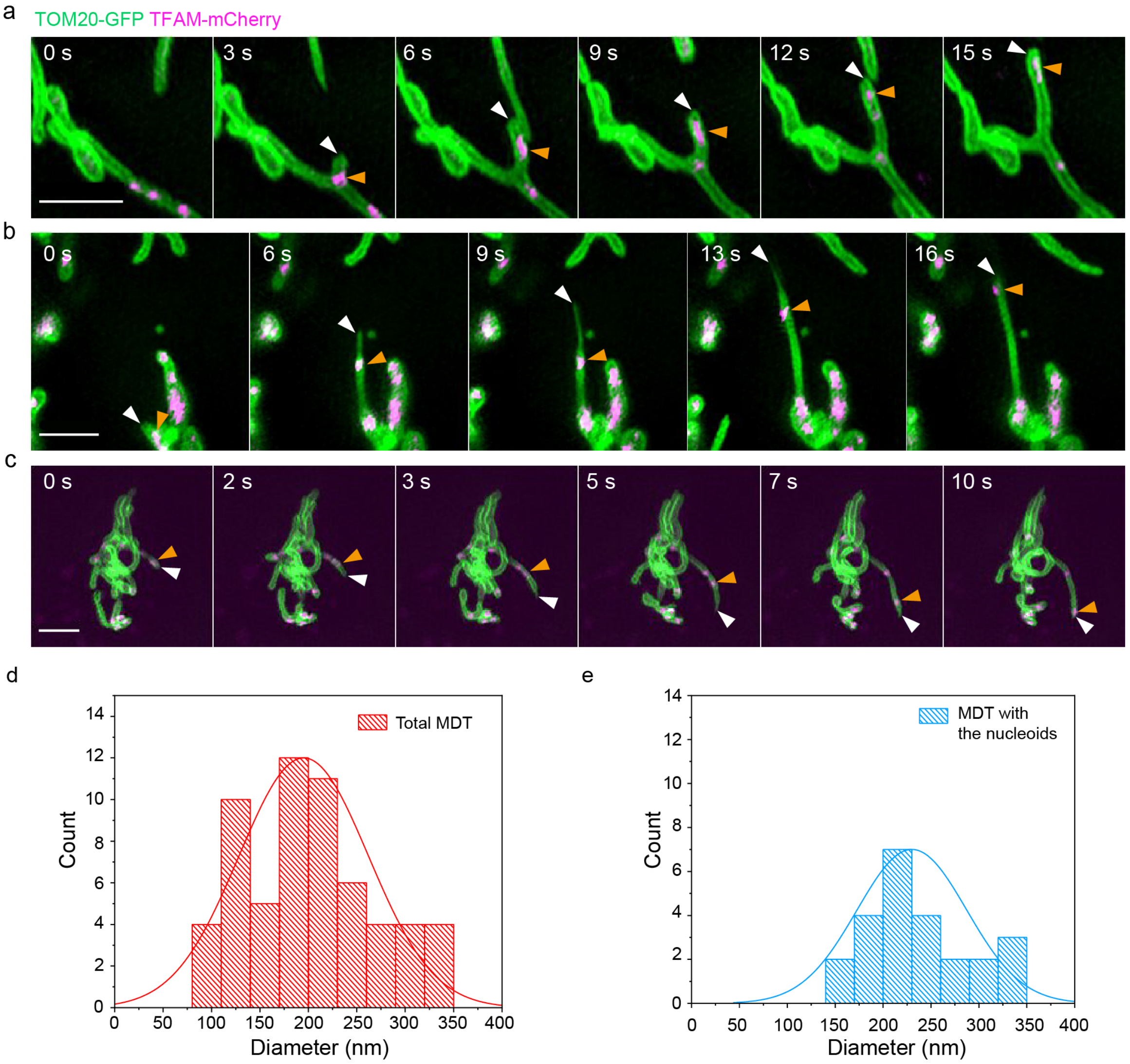

f

MDT categorization	The percentage of nucleoids-containing MDT
MDT diameter ≤ 200nm	22% (8/36)
MDT diameter > 200 nm	70% (16/23)

Supplementary Fig. 2 Mitochondrial DNA nucleoids are transported via MDT. a-c) Additional examples of MDT events during indicated time courses in Cos-7 cells expressing Tom20-GFP and TFAM-mCherry show that mitochondrial DNA nucleoids are transported via MDT. White arrowheads mark the tips of tubules generated by the MDT process. Orange arrowheads indicate the TFAM-mCherry–labeled mtDNA nucleoids. Scale bar: 2 μm. **d)** The diameter distribution of total MDT, n= 59 events from 10 cells. **e)** The diameter distribution of MDT with the nucleoids, n= 29 events from 10 cells. **f)** Percentage of nucleoids-containing tubules in different sizes of MDT. n=36 for MDT with diameter ≤ 200 nm, n=23 for MDT with diameter > 200nm. Source data are provided as a Source Data file.

a

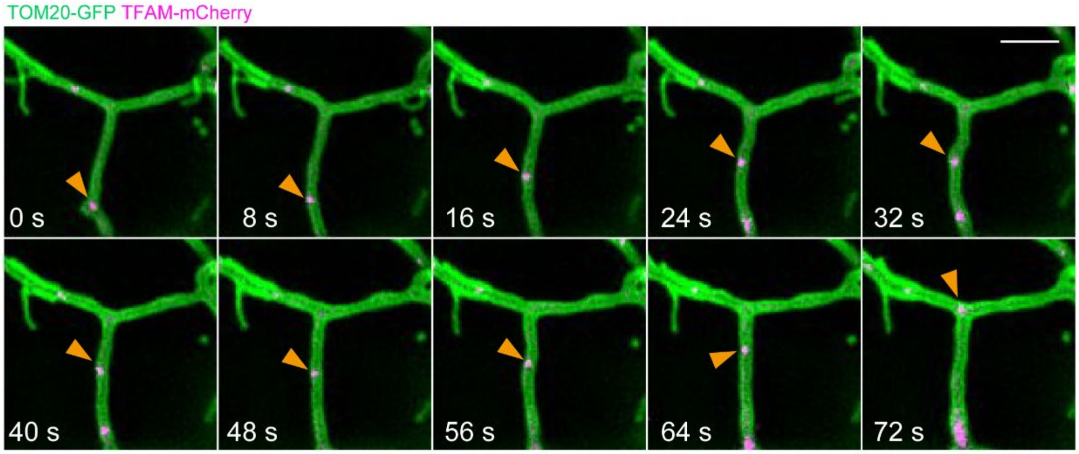

b

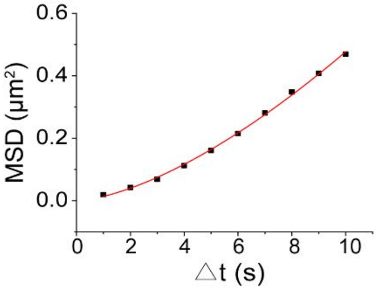

Supplementary Fig. 3 Active transportation of the nucleoid in static mitochondrial network. a) Time-lapse images of a nucleoid being transported in the mitochondrial network of a Cos-7 cell. Arrowheads indicate the nucleoid. Scale bar: 2μm. **b)** Mean squared displacement (MSD) of the nucleoid in **a**. Source data are provided as a Source Data file.

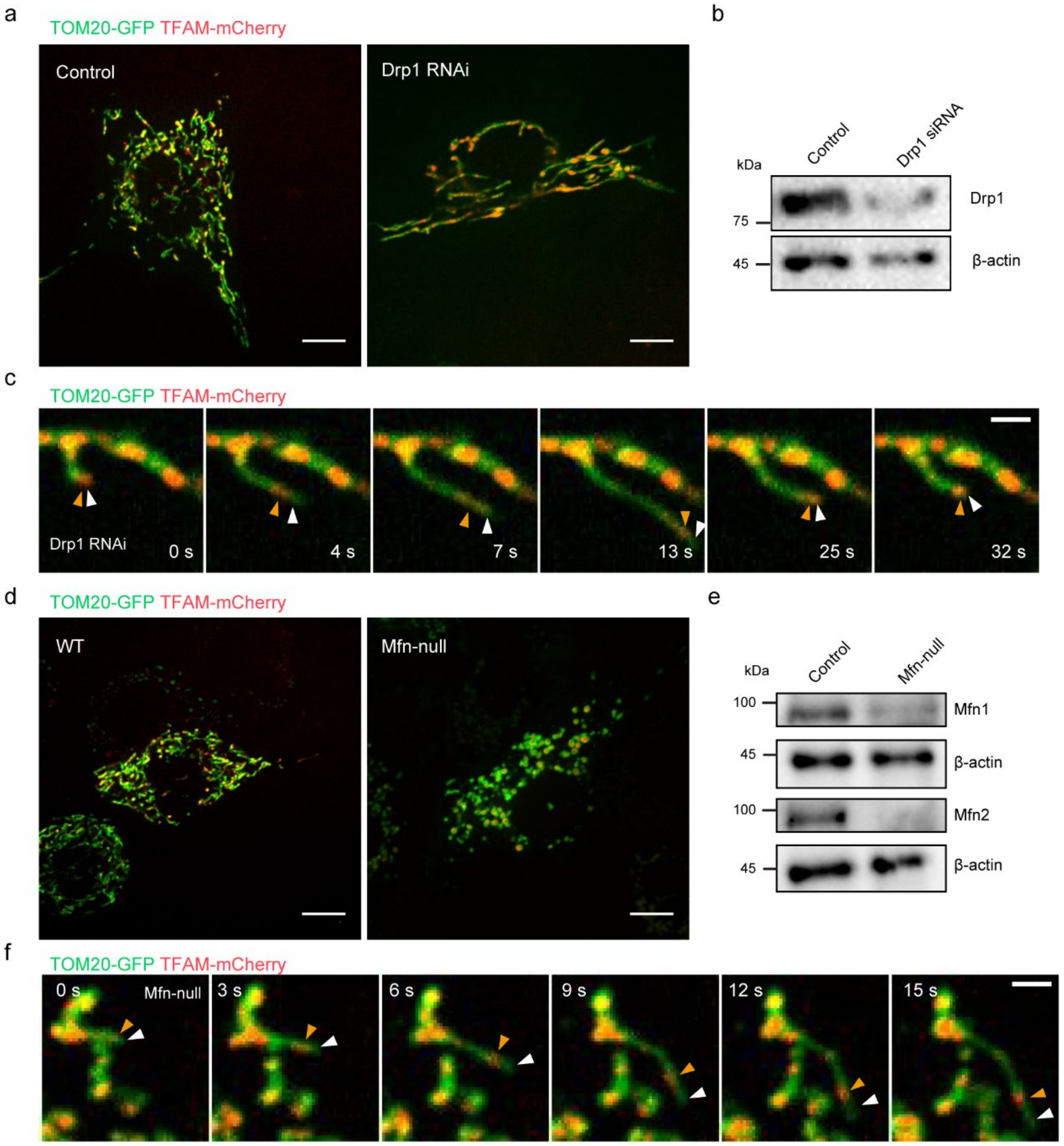

Supplementary Fig. 4 MDT-based nucleoids transport is independent on mitochondrial fission and fusion. a) Control and Drp1 siRNA MEF cells expressing TOM20-GFP and TFAM-mCherry. Scale bar: 10 μm. **b)** Western blots of Drp1 indicating Drp1 depletion in lysates of cells transfected with siRNA against Drp1. **c)** Time-lapse sequence of MDT-based nucleoids transport in Drp1 knockdown MEF cells. White arrows mark the tips of tubules generated by MDT. Orange arrows indicate the nucleoids. Scale bar, 2 μm. **d)** Control and Mfn-null MEF cells expressing TOM20-GFP and TFAM-mCherry. Scale bar, 10 μm. **e)** Mfn1 and Mfn2 levels in Mfn-null and control MEF cells shown by western blotting. **f)** Time-lapse sequence of MDT-based nucleoids transport in Mfn-null MEF cells. White arrows mark the tips of tubules generated by MDT. Orange arrows indicate nucleoids. Scale bar, 2 μm.

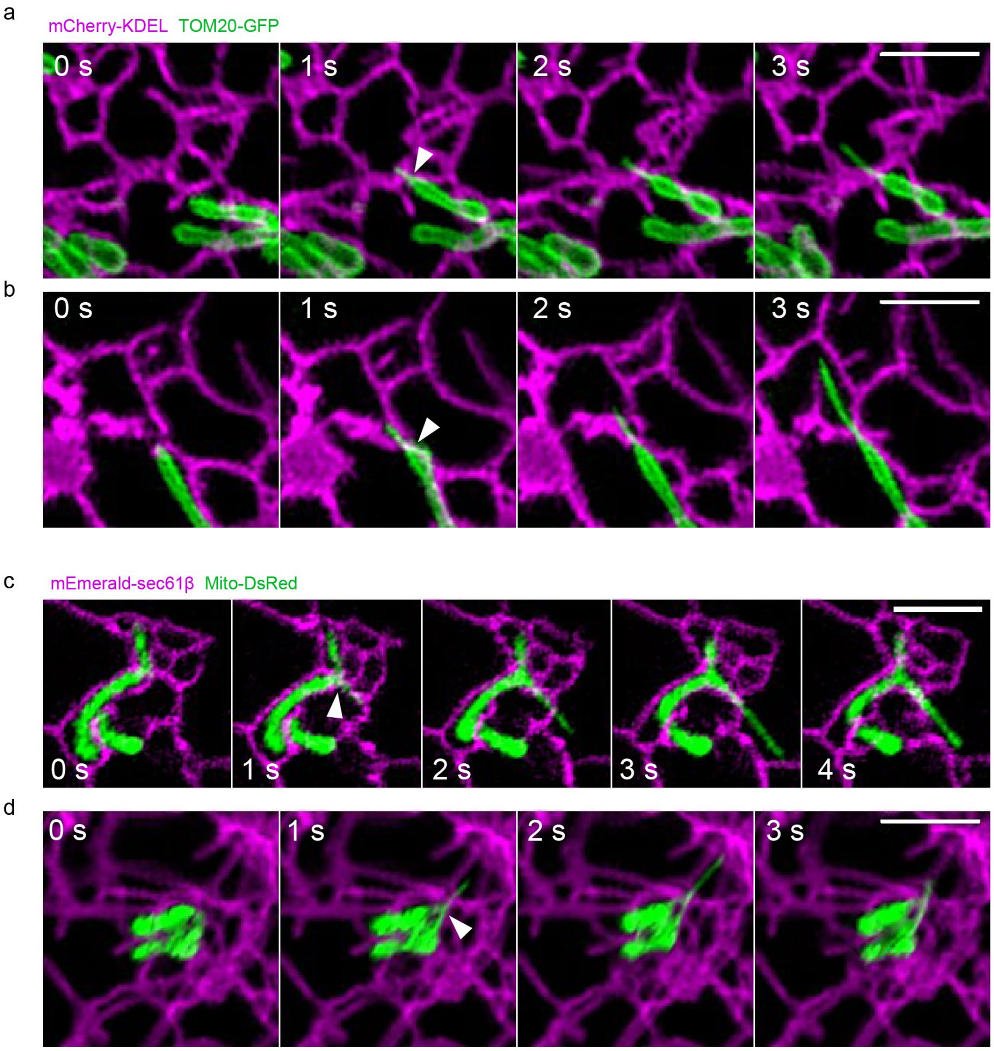

e

MDT categorization	ER tubules mark the initiation sites of MDT
MDT diameter ≤ 200nm	92% (24/26)
MDT diameter > 200 nm	88% (22/25)

Supplementary Fig. 5 ER tubules mark the initiation site of MDT. a-d) Additional examples of MDT events (as in Fig. 2) during indicated time courses in Cos-7 cells expressing Tom20-GFP and mCherry-KDEL, or mito-DsRed and mEmerald-sec61β, show that MDT occurs predominantly at positions where an ER tubules cross over the mitochondria. Arrowheads indicate the MDT initiation sites. Scale bar: 2 μm. **e)** Percentage of MDT with different tubular diameters in live Cos-7 cells that occurred at the EMCS. n=26 for MDT with diameter ⩽ 200nm, n=25 for MDT with diameter > 200nm. Source data are provided as a Source Data file.

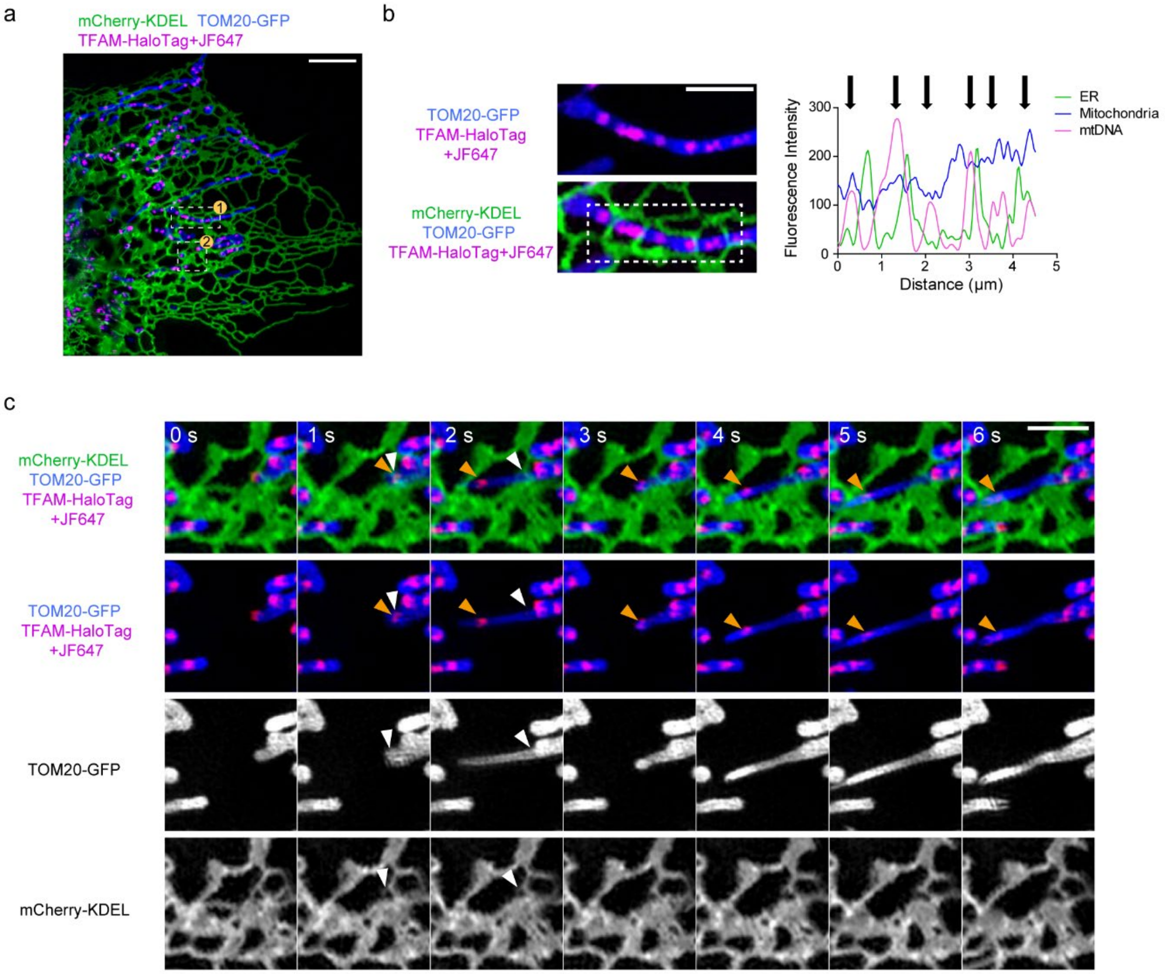

Supplementary Fig. 6 Mitochondrial DNA nucleoids are transported via MDT at the EMCS. a) A merged image of a live Cos-7 cell expressing Tom20-GFP, TFAM-HaloTag (labeled with JF647 ligand, i.e. TFAM-JF647), and mCherry-KDEL. Scale bar: 5 μm. **b)** (Left) An example shows mitochondrial DNA nucleoids are spatially linked to EMCS (white box 1 in **a**); (Right) The pixel intensity of Tom20-GFP, TFAM-JF647 and mCherry-KDEL from a linescan drawn along the mitochondrial tubule (in the dashed box). Black arrows indicate the nucleoid positions. Scale bar: 2 μm. **c)** Examples of mitochondrial DNA nucleoids at the EMCS transported by MDT (white box 2 in **a**). White arrowheads indicate the initiation site of MDT and orange arrowheads indicate the nucleoid. Scale bar: 2 μm.

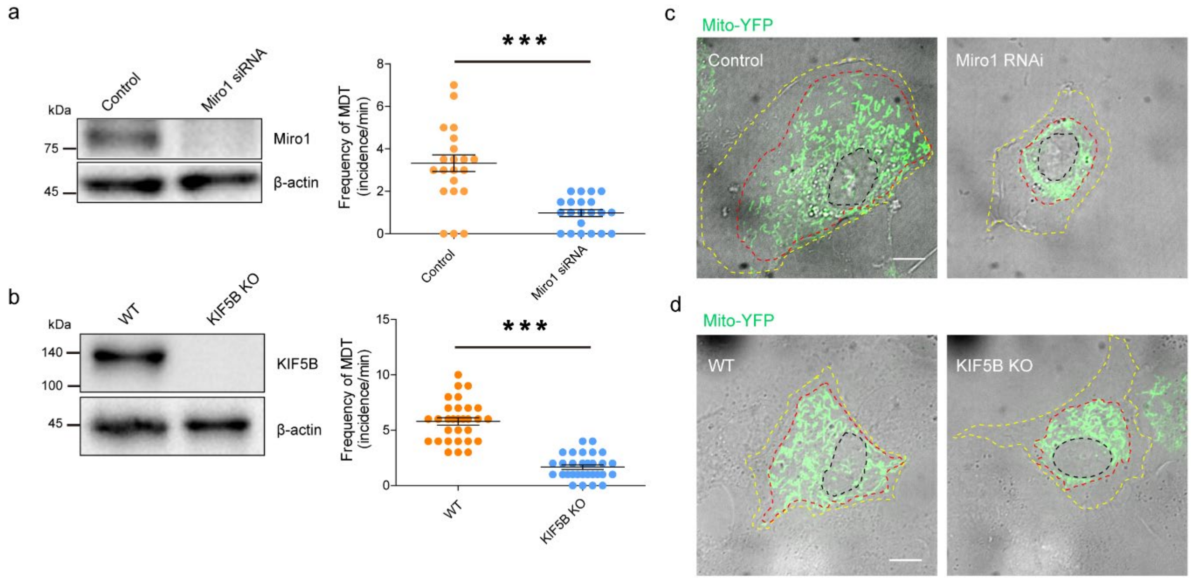

Supplementary Fig. 7 Miro1 is a receptor of KIF5B on mitochondria. a) Left panel, western blots of Miro1 and β-actin demonstrate depletion of Miro1 in lysates from cells transfected with siRNA against Miro1 and with control siRNA cells. Right panel, MDT frequency ratio for the control and Miro1 RNAi cells (n = 21 cells were used per condition from three independent experiments. Data are presented as mean ± SEM. $p < 0.0001$, ***$p < 0.001$, two-tailed, unpaired Student's *t* test). **b)** Left panel, knockout of KIF5B in cells. Expression levels of KIF5B were examined by western blotting. Right panel, MDT frequency ratio for the WT and KIF5B-knockout cells (n = 30 cells were used per condition from three independent experiments. Data are presented as mean ± SEM. $p < 0.0001$, ***$p < 0.001$, two-tailed, unpaired Student's *t* test). **c)** The control and Miro1-knockdown cells expressing mito-YFP were observed by spinning-disk microscopy. Yellow dashed lines indicate the cell boundary; red dashed lines indicate the boundary of the mitochondrial network; and black dashed lines indicate the boundary of nucleus. Scale bar: 10 µm. **d)** The WT and KIF5B-knockout cells expressing mito-YFP were observed by spinning-disk microscopy. Yellow dashed lines indicate the cell boundary; red dashed lines indicate the boundary of the mitochondrial network; and black dashed lines indicate the boundary of nucleus. Scale bar: 10 µm. Source data are provided as a Source Data file.

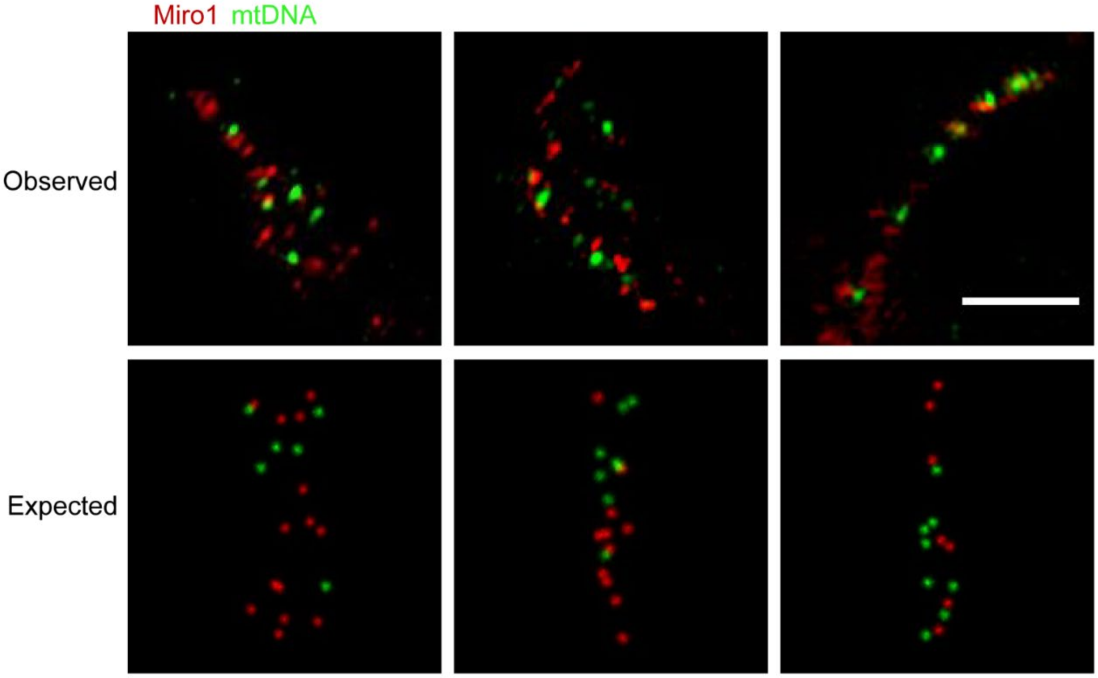

Supplementary Fig. 8 Miro1 is spatially associated with mtDNA. Top row shows the data presented in Fig. 3d. Green represents mtDNA and red indicates Miro1. Bottom row is a Matlab simulation, showing randomly scrambled distribution of mtDNA and Miro1 foci of the observed data. Scale bar: 2 µm.

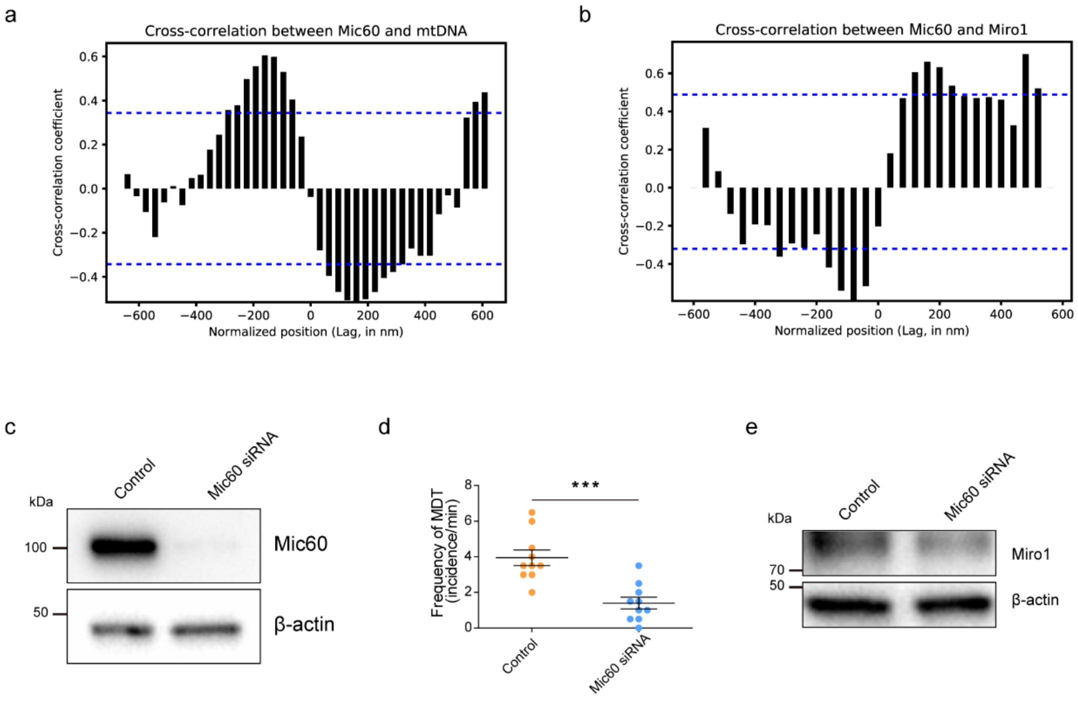

Supplementary Fig. 9 Mic60 is spatially associated with mtDNA and Miro1. a) Spatial cross-correlation analysis of mtDNA and Mic60 fluorescence intensity along linescans of mitochondria (n = 32) in fixed Cos-7 cells. Blue dotted lines indicate 95% confidence interval cut-offs. **b)** Spatial cross-correlation analysis of Miro1 and Mic60 fluorescence intensity along linescans of mitochondria (n = 30) in fixed Cos-7 cells. Blue dotted lines indicate 95% confidence interval cut-offs. **c)** Western blots of Mic60 indicate depletion of Mic60 in lysates of cells transfected with siRNA against Mic60. **d)** MDT frequency of the control and Mic60 RNAi cells (n=10 cells were used per condition from three independent experiments). Data are presented as mean ± SEM. $p = 0.0002$, ***$p < 0.001$, two-tailed, unpaired Student's *t* test. **e)** Western blots of Miro1 indicate decreased expression level of Miro1 in Mic60-knockdown Cos-7 cells. With β-actin normalization, the analysis showed that the reduction of Miro expression upon Mic60 knockdown was 41% in comparison with the control cells. Source data are provided as a Source Data file.

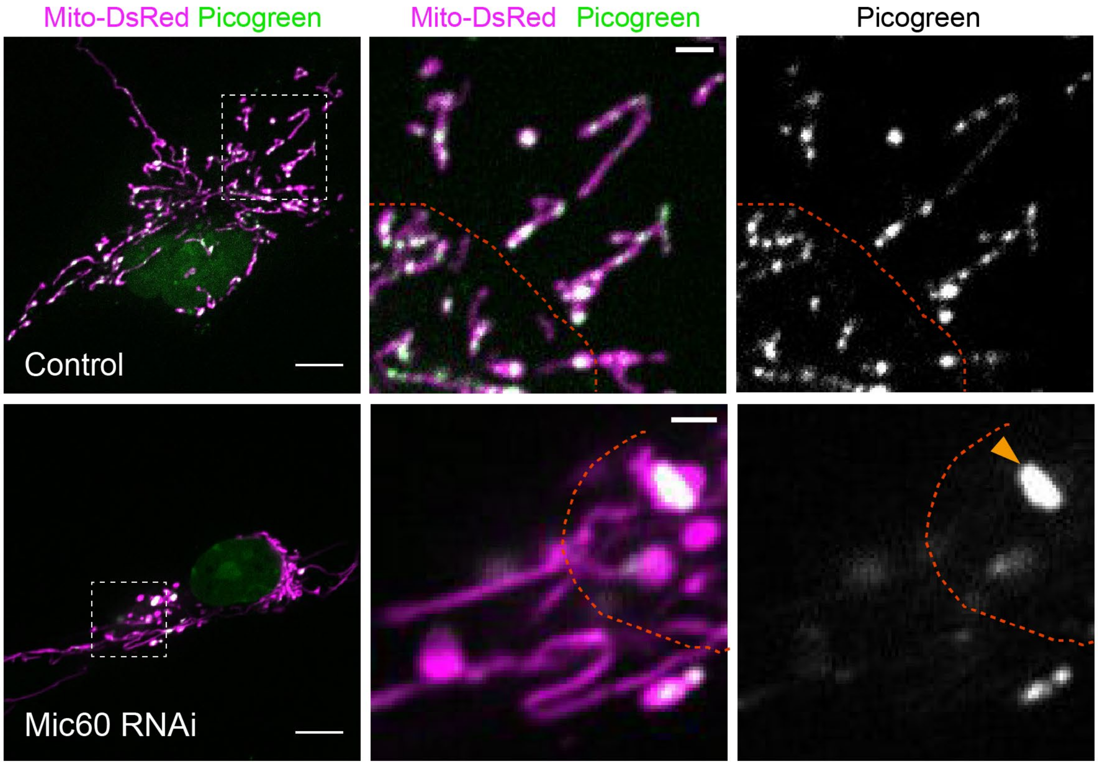

Supplementary Fig. 10 MDT contributes to mtDNA distribution in the Cos-7 cell. Mic60 RNAi and control Cos-7 cells expressing mito-DsRed and stained by picogreen. Scale bar: 10 µm. Boxed areas in the first 2 images are magnified and presented as merged channel and mtDNA channel, respectively. The orange arrow indicates an enlarged mtDNA. Red dashed lines indicate the boundary of the perinuclear mitochondria. Scale bar: 2 µm.

Supplementary Table 1. Primers used in this study.

Name	Sequence
Mito-BFP-Fw	CGCGGATCCGGTCTCACCGGTCGCCACCATGAG
Mito-BFP-Rv	AAGGAAAAAAGCGGCCGCGGTCTCTTAGTGCCCCAGTTTGCTAGGGA
TFAM-GFP-Fw	ccggaattcGGTCTCATGGCGTTTCTCCGAAGC
TFAM-GFP-Rv	cgcggatccaaGGTCTCACACTCCTCAGCACCATATTTT
TFAM-mCherry-Fw	CGCggatccaccggtcgccaccATGGTGAGCAAGGGCGAGGA
TFAM-mCherry-Rv	AAGGAAAAAAGCGGCCGCTTTACTTGTACAGCTCGTCCATGCCG
