# Supplementary material for: ER-mitochondria contacts promote mtDNA nucleoids active transportation via mitochondrial dynamic tubulation
Source: Nat Commun. 2020 Sep 8;11:4471. doi: 10.1038/s41467-020-18202-4 (PMC7478960; doi:10.1038/s41467-020-18202-4)
Supplement: Supplementary file 9 — Description of Additional Supplementary Files [file 41467_2020_18202_MOESM9_ESM.pdf]

Title: Supplementary Movie 1 MDT are spatially linked to Mitochondrial DNA nucleoids.
Description: Cos-7 cells expressing TOM20-GFP and TFAM-mCherry were visualized with a GI-SIM system. Scale bar, 1 μm.

Title: Supplementary Movie 2 Mitochondrial DNA nucleoids are transported via MDT.
Description: Cos-7 cells expressing TOM20-GFP and TFAM-mCherry were visualized with a GI-SIM system. White arrowheads indicate the tubulation process. Yellow arrowheads indicate the sites of nucleoids. Scale bar, 2 μm.

Title: Supplementary Movie 3 Active transportation of the mitochondrial nucleoid within mitochondria.
Description: Cos-7 cells expressing TOM20-GFP and TFAM-mCherry were visualized with a GI-SIM system. Yellow arrowhead indicate the site of the nucleoid. Scale bar, 2 μm.

Title: Supplementary Movie 4 ER tubules mark the initiation sites of MDT.
Description: Cos-7 cells expressing Mito-DsRed and mEmerald-sec61β (first row), or Tom20-GFP and mCherry-KDEL (second row), were visualized with a GI-SIM system. White arrowheads indicate the tubulation process. Yellow arrowheads indicate the initiation sites of MDT. Scale bar, 1 μm.

Title: Supplementary Movie 5 Mitochondrial DNA nucleoids are transported via MDT at the EMCS.
Description: Left panel: Cos-7 cell expressing Tom20-GFP, TFAM-Halo-tag, mCherry-KDEL and labeled with JF647 ligand. Scale bar: 5μm. Right panel: 2 An example of mitochondrial DNA nucleoids at ER contact sites transported by MDT. White arrowheads indicate the tubulation process. Yellow arrowheads indicate the sites of nucleoids. Scale bar: 2μm.

Title: Supplementary Movie 6 Active transportation of mtDNA is inhibited in Mic60 KD cells.
Description: Cos-7 cells expressing Mito-DsRed and stained by picogreen were visualized with a GI-SIM system. mtDNA movement were visualized in a control cell and Mic60 RNAi cell. White arrowheads indicate the sites of mtDNA. Scale bar, 2 μm.
