# Supplementary material for: ER-mitochondria contacts promote mtDNA nucleoids active transportation via mitochondrial dynamic tubulation
Source: Nat Commun. 2020 Sep 8;11:4471. doi: 10.1038/s41467-020-18202-4 (PMC7478960; doi:10.1038/s41467-020-18202-4)
Supplement: Supplementary file 10 — Reporting Summary [file 41467_2020_18202_MOESM10_ESM.pdf]

nature research

Corresponding author(s): Yujie Sun

Last updated by author(s): Jul 9, 2020

Reporting Summary

Nature Research wishes to improve the reproducibility of the work that we publish. This form provides structure for consistency and transparency in reporting. For further information on Nature Research policies, see Authors & Referees and the Editorial Policy Checklist.

Statistics

For all statistical analyses, confirm that the following items are present in the figure legend, table legend, main text, or Methods section.

n/a	Confirmed	
☐	☒	The exact sample size (*n*) for each experimental group/condition, given as a discrete number and unit of measurement
☐	☒	A statement on whether measurements were taken from distinct samples or whether the same sample was measured repeatedly
☐	☒	The statistical test(s) used AND whether they are one- or two-sided<br>*Only common tests should be described solely by name; describe more complex techniques in the Methods section.*
☒	☐	A description of all covariates tested
☒	☐	A description of any assumptions or corrections, such as tests of normality and adjustment for multiple comparisons
☐	☒	A full description of the statistical parameters including central tendency (e.g. means) or other basic estimates (e.g. regression coefficient) AND variation (e.g. standard deviation) or associated estimates of uncertainty (e.g. confidence intervals)
☐	☒	For null hypothesis testing, the test statistic (e.g. *F*, *t*, *r*) with confidence intervals, effect sizes, degrees of freedom and *P* value noted<br>*Give P values as exact values whenever suitable.*
☒	☐	For Bayesian analysis, information on the choice of priors and Markov chain Monte Carlo settings
☒	☐	For hierarchical and complex designs, identification of the appropriate level for tests and full reporting of outcomes
☐	☒	Estimates of effect sizes (e.g. Cohen's *d*, Pearson's *r*), indicating how they were calculated

Our web collection on statistics for biologists contains articles on many of the points above.

Software and code

Policy information about availability of computer code

Data collection	Labview (version 2016) and Volocity (version 6.3) were used for image acquisition. Image lab (version 5.0) was used for Western blotting images.
Data analysis	FIJI (ImageJ, version 2.0) was used for image analysis and export. Image Lab (version 5.0) was used for exporting Western blotting images. Prism (version 5.0) and Origin (version 2018b) were used for mathematical analysis and graphing of microscopy data. Python (version 2.7) and MATLAB (version R2018a) were used for colocalization analysis. All code used in this study have been deposited in Zenodo (DOI: 10.5281/zenodo.3939035).

For manuscripts utilizing custom algorithms or software that are central to the research but not yet described in published literature, software must be made available to editors/reviewers. We strongly encourage code deposition in a community repository (e.g. GitHub). See the Nature Research guidelines for submitting code & software for further information.

Data

Policy information about availability of data

All manuscripts must include a data availability statement. This statement should provide the following information, where applicable:

- Accession codes, unique identifiers, or web links for publicly available datasets
- A list of figures that have associated raw data
- A description of any restrictions on data availability

The raw data generated in this study are available in Zenodo (DOI: 10.5281/zenodo.3939035). All other data supporting the findings of this study are available within the article and its Supplementary information files or from the corresponding authors upon reasonable request. The source data underlying Figs. 1b, 1d, 1e, 3c, 3e, 3f, 4e, 4h, 5g and 5j and Supplementary Figs. 1c, 2d, 2e, 2f, 3b, 5e, 7a, 7b, 9a, 9b and 9d are provided as a Source Data file. Source data are provided with this paper.

Field-specific reporting

Please select the one below that is the best fit for your research. If you are not sure, read the appropriate sections before making your selection.

☒ Life sciences ☐ Behavioural & social sciences ☐ Ecological, evolutionary & environmental sciences

For a reference copy of the document with all sections, see nature.com/documents/nr-reporting-summary-flat.pdf

Life sciences study design

All studies must disclose on these points even when the disclosure is negative.

Sample size	No statistical method is used to predetermine the sample size. Sample size was determined by experimental factors. For microscopy experiments, 10-30 cells per condition were imaged and 15-100 events were collected. For western blotting, 3 immunoblots were performed following normal practice in the field.
Data exclusions	No data were excluded from the analyses.
Replication	All attempts at replication were successful in all experiments. Each experiment was repeated three times independently with similar results.
Randomization	Samples were allocated randomly to experimental group.
Blinding	Not applicable. For example, Miro1 KD cells have a strong mitochondrial phenotype, which make investigators identify the experimental group.

Reporting for specific materials, systems and methods

We require information from authors about some types of materials, experimental systems and methods used in many studies. Here, indicate whether each material, system or method listed is relevant to your study. If you are not sure if a list item applies to your research, read the appropriate section before selecting a response.

Materials & experimental systems

n/a	Involved in the study	
☐	☒	Antibodies
☐	☒	Eukaryotic cell lines
☒	☐	Palaeontology
☒	☐	Animals and other organisms
☒	☐	Human research participants
☒	☐	Clinical data

Methods

n/a	Involved in the study	
☒	☐	ChIP-seq
☒	☐	Flow cytometry
☒	☐	MRI-based neuroimaging

Antibodies

Antibodies used

Anti-Miro1, mouse monoclonal (WH0055288M1; Sigma-Aldrich); anti-Miro1, rabbit polyclonal (PA5-72835; Invitrogen); anti-DNA, mouse monoclonal (AC-30-10, Progen); anti-Mitofilin, rabbit polyclonal (10179-1-AP, Proteintech); anti-KIF5B, rabbit monoclonal (ab167429, Abcam); anti-Mitofusin1, rabbit monoclonal (ab57602, Abcam); anti-Mitofusin2, rabbit monclonal (ab124773, Abcam); anti-DRP1, rabbit monoclonal (ab184247, Abcam); rabbit anti-TFAM (HPA040648; Sigma-Aldrich); anti-β actin, mouse monoclonal (sc-47778, Santa Cruz Biotechnology); horseradish peroxidase-conjugated Goat anti–Rabbit (7074S, Cell Signaling Technology); Goat anti–Mouse secondary antibody, HPR (31436, Invitrogen).

Validation

Anti-Miro1, mouse monoclonal (WH0055288M1; Sigma-Aldrich). Validation with western blot analysis of Hela S3 NE. Validation with immunofluorescence analysis of Cos-7 cells in published papers cited below. Species reactivity: Human. Applications: indirect ELISA (suitable); western blot (1-5 μg/mL).https://www.sigmaaldrich.com/catalog/product/sigma/wh0055288m1?lang=zh&region=CN

1. Kornmann B, Osman C, Walter P. The conserved GTPase Gem1 regulates endoplasmic reticulum-mitochondria connections. Proc. Natl. Acad. Sci. U. S. A. 108, 14151-14156 (2011).

anti-Miro1, rabbit polyclonal (PA5-72835; Invitrogen).Validation with western blot analysis of rat brain tissue lysate. Species reactivity: Human, Mouse, Rat. Applications: ELISA (Assay-dependent); western blot (1-2 μg/mL);ICC (Assay-dependent); IF: 20 μg/mL; IHC (P): 5 μg/mL. https://www.thermofisher.com/cn/zh/antibody/product/RHOT1-Antibody-Polyclonal/PA5-72835

anti-DNA, mouse monoclonal (AC-30-10, Progen).Validation with mmunofluorescence analysis of Cos-7 cells in our specific experiments. Species reactivity: All Species. Applications: Immunohistochemistry (Frozen Sections) (IHC (fro)), Immunohistochemistry (Paraffin-embedded Sections) (IHC (p)) https://www.antibodies-online.com/antibody/284371/anti-Deoxyribonucleic+Acid+DNA+antibody/

anti-Mitofilin, rabbit polyclonal (10179-1-AP, Proteintech). Validation with western blot analysis of multi-cells/tissue, COLO 320 cells, HEK-293 cells, HeLa cells, HepG2 cells, human brain tissue, MCF-7 cells, Raji cells. Validation with IF analysis of Hela cells. Validation with IP analysis of Raji cells. Species reactivity: human, mouse, hamster, rat. WB (1:500-1:2000); IP (0.5-4.0 ug); IF (1:50-1:500); IHC (1:20-1:200). https://www.ptgcn.com/Products/IMMT-Antibody-10179-1-AP.htm

anti-KIF5B, rabbit monoclonal (ab167429, Abcam). Validation with western blot analysis of Jurkat, Hela, Mouse brain lysates and Rat brain lysates. Species reactivity: human, mouse, rat. Applications: WB (1:1000-1:10000); IP (1:10-1:100); IHC-P (1:100-1:250); IF (1/100-1/500). https://www.abcam.com/kif5b-antibody-epr10276b-ab167429.html#description_images_1

anti-Mitofusin1, rabbit monoclonal (ab57602, Abcam). Validation with western blot analysis of Mouse cardiomyocytes whole cell lysate and Hela cell lysate. Species reactivity: Mouse, Rat, Human, Cynomolgus monkey. Applications: WB, IHC-P, ICC/IF, Flow Cyt, IP. https://www.abcam.com/mitofusin-2--mitofusin-1-antibody-3c9-ab57602.html#description_images_3

anti-Mitofusin2, rabbit monclonal (ab124773, Abcam). Validation with western blot analysis of mouse brain lysate, mouse kidney lysate and rat brain lysate. Species reactivity: Mouse, Rat, Human. Applications: WB (1:1000-1:10000), IHC-P (1:50-1:100), ICC/IF (1:300). https://www.abcam.com/mitofusin-2-antibody-niar164-ab124773.html#description_images_1

anti-DRP1, rabbit monoclonal (ab184247, Abcam). Validation with western blot analysis of A549, U2OS, Hela, Jurkat, HEK-293 and HCT 116. Species reactivity: Mouse, Rat, Human. Applications: WB (1:1000), ICC/IF (1:250), IP (1:30), Flow Cyt (1:70), IHC-P (1:1000). https://www.abcam.com/drp1-antibody-epr19274-ab184247.html#description_images_1

anti-TFAM, rabbit polyclonal (HPA040648; Sigma-Aldrich). Validation with western blot analysis of human cell lines Caco-2 and Hela. Species reactivity: Human. Applications: WB (0.04-0.4 μg/mL), IF (0.25-2 μg/mL), IHC (1:50-1:200). https://www.sigmaaldrich.com/catalog/product/sigma/hpa040648?lang=zh&region=CN

anti-β actin, mouse monoclonal (sc-47778, Santa Cruz Biotechnology). Validation with western blot analysis of HeLa, Sol8, C32 and NIH/3T3 whole cell lysates. Species reactivity: mouse, rat, human, avian, bovine, canine, porcine, rabbit, Dictyostelium discoideum and Physarum polycephalum origin. Applications: WB (1:100-1:1000), IHC(P) (1:50-1:500) and ELISA (1:30-1:3000). https://www.scbt.com/p/beta-actin-antibody-c4?requestFrom=search

Eukaryotic cell lines

Policy information about cell lines

Cell line source(s)	Cos-7, NRK, MEF and HEK293T are obtained from the American Type Culture Collection (ATCC). Generation of normal rat kidney cells with a stable knockout of KIF5B or with tetracycline-inducible expression of KIF5B were gifts from Dr. Li Yu (Tsinghua University).
Authentication	Cos-7 cells, MEF cells and HEK293T cells were not authenticated. NRK cells were previously authenticated by Western blotting and reported by our group in detail.
Mycoplasma contamination	All cell lines were tested negative for mycoplasma contamination.
Commonly misidentified lines (See ICLAC register)	We have not used any commonly misidentified line in our study.
